# Antimalarial drugs—are they beneficial in rheumatic and viral diseases?—considerations in COVID-19 pandemic

**DOI:** 10.1007/s10067-021-05805-5

**Published:** 2021-07-03

**Authors:** Bogna Grygiel-Górniak

**Affiliations:** Department of Rheumatology, Rehabilitation and Internal Medicine, Poznan University of Medical Sciences, Poznan, Poland

**Keywords:** Antimalarial drugs, COVID-19, Rheumatic diseases, Viral diseases

## Abstract

The majority of the medical fraternity is continuously involved in finding new therapeutic schemes, including antimalarial medications (AMDs), which can be useful in combating the 2019-nCoV: coronavirus disease (COVID-19). For many decades, AMDs have been widely used in the treatment of malaria and various other anti-inflammatory diseases, particularly to treat autoimmune disorders of the connective tissue. The review comprises in vitro and in vivo studies, original studies, clinical trials, and consensus reports for the analysis, which were available in medical databases (e.g., PubMed). This manuscript summarizes the current knowledge about chloroquine (CQ)/hydroxychloroquine (HCQ) and shows the difference between their use, activity, recommendation, doses, and adverse effects on two groups of patients: those with rheumatic and viral diseases (including COVID-19). In the case of connective tissue disorders, AMDs are prescribed for a prolonged duration in small doses, and their effect is observed after few weeks, whereas in the case of viral infections, they are prescribed in larger doses for a short duration to achieve a quick saturation effect. In rheumatic diseases, AMDs are well tolerated, and their side effects are rare. However, in some viral diseases, the effect of AMDs is questionable or not so noticeable as suggested during the initial prognosis. They are mainly used as an additive therapy to antiviral drugs, but recent studies have shown that AMDs can diminish the efficacy of some antiviral drugs and may cause respiratory, kidney, liver, and cardiac complications.

## Introduction

Antimalarial drugs (AMDs) have been used in the treatment of malaria and other inflammatory diseases for over 70 years. Chloroquine (CQ) was first synthesized AMD in Germany by Bayer in 1934. It was used as a synthetic replacement for natural quinine in the treatment of malaria [1]. However, due to drug resistance, CQ is less often implemented in the treatment of malaria.

Over the last decades, the anti-inflammatory and immunomodulatory properties of AMDs became the main reason for their use in rheumatic diseases [2]. They are recommended in the treatment of rheumatoid arthritis (RA), systemic lupus erythematosus (SLE), sarcoidosis, dermatomyositis, Sjögren’s syndrome, chronic juvenile arthritis, psoriatic arthritis, and various other autoimmune diseases, because of their good efficacy and acceptable safety profiles [3]. AMDs are advantageous because of their excellent safety profile (especially hydroxychloroquine (HCQ)). These medications reduce skin lesions exacerbated by ultraviolet light, arthralgia, and myalgia, improve fatigue, decrease disease activity, and diminish cardiovascular risk [4–8]. They have immunomodulatory, antiviral, antitumor, and antithrombotic effects and play a beneficial role in the regulation of metabolism [3].

Recently, AMDs have been used to treat many other viral diseases. The earlier studies of AMDs as antiviral agents were not very encouraging (e.g., in Chikungunya virus) [9], and the early reports indicated that AMDs had no effect on viral diseases in humans [10, 11]. More recent data show some efficacy of AMDs in HIV and novel severe acute respiratory syndrome coronavirus 2 (SARS-CoV-1). However, initially, the treatment of 2019-nCoV: coronavirus disease (COVID-19) with AMDs was met with skepticism, and they were not recommended in quickly spreading viral infections [12]. Nevertheless, chloroquine (CQ) and HCQ were approved for the treatment of COVID-19 based on their in vitro antiviral activity [13]. The decision of using AMDs in the treatment of COVID-19 was also supported by the anti-inflammatory activity and inhibition of the production of proinflammatory cytokines, such as tumor necrosis factor (TNF)-α and interleukin (IL)-6. This effect is named a “cytokine storm” and is reduced by AMDs [14, 15]. Subsequent studies and clinical experience have underlined the efficacy and the beneficial effects of AMDs in patients with COVID-19, which is primarily based on observational studies in small groups [16, 17]. Later analysis has shown the adverse effect of AMDs in COVID-19 treatment, which caused an increased risk of cardiovascular complications, and even death in severely infected patients [18, 19].

Given the fact that AMDs are extensively used in rheumatic and viral diseases, in this review, we discuss the mechanism, indications, recommended or suggested doses, efficiency, and adverse effects of AMDs. We also present a critical point of view and the latest evidence of the beneficial or harmful effects of AMDs in various clinical disorders, underling their impact on SARS-CoV-2 infection.

## AMDs—properties and mechanism of action in rheumatic diseases

AMDs are lipophilic drugs and have weak alkaline properties. They inhibit lysosomal degradation/autophagy either by altering lysosomal acidification or by inhibiting the levels of lysosomal-associated membrane proteins (LAMPs) [20]. AMDs encompass three drugs: CQ, HCQ, and quinacrine; however, CQ and HCQ are most often used in rheumatic and viral diseases [21]. HCQ differs from CQ in that it contains a hydroxyl group at the end of the side chain. Both CQ and HCQ show similar pharmacokinetic behavior and are quickly absorbed by the intestine and excreted by the kidneys. Moreover, their recommended doses in rheumatic diseases are well tolerated and have less toxicity [15].

AMDs are particularly useful in skin conditions exacerbated by light (hypersensitivity associated with ultraviolet light). However, the pleiotropic action of AMDs shows a broad spectrum of activity on coagulation and the levels of serum lipid, and glucose resulting in a decreased risk of cardiovascular diseases (CVDs) [5, 17]. Compared with other chronically used immunosuppressants (e.g., methotrexate (MTX), leflunomide (LF), or cyclophosphamide), the use of CQ and HCQ is associated with a reduced risk of infection [22]. Therefore, AMDs are commonly proposed for patients with rheumatic diseases who have mainly viral and bacterial infections and who have comorbidities (Table 1).

**Table 1:** The use of antimalarial drugs (AMDs) in rheumatic diseases

AMDs in rheumatic diseases			
Analyzed disease	Drug appliance	Mechanism of drugs action	Clinical effect
Systemic lupus erythematosus including SCLE, DLE, and LEP	First-line drugs HCQ sulfate CQ phosphate	have immunomodulatory potency interfere with lysosomal activity and autophagy, alter signaling pathways and transcriptional activity = > inhibition of cytokine production and modulation of specific co-stimulatory molecules [23] CQ and HCQ change the pH of lysosomes, where toll-like receptor (TLR)-7 and TLR9 are located = > ↓ the binding affinity of ds-DNA immune complexes to TLR9 and downregulates interferon production in SLE [24].	↓ Fatigue and general weakness ↓ The activity of the disease [4] ↓ Organ damage [25] and reduce the infection rate [4] Improve cutaneous and musculoskeletal symptoms (↓ ulcers of mucous membranes and ↓ pain of joints and muscles) [25] Protect against thrombosis and bone mass loss [4, 26] Prevent complications of the central nervous system lupus [26] ↓ Renal involvement and ↓ progression of kidney damage in SLE [27, 28] ↓ The incidence of serious inflammation (e.g., cardiac and pleura involvement) Have antithrombotic effects and prevent venous thromboembolism in patients with SLE and APS [26] Reveal cardiovascular protection due to hypoglycemic and lipid-lowering effects [5] Enable to lower the doses of GCS [4] Prevent lupus flares [4, 27] Reduce the risk of pregnancy complications [29] Increase the long-term survival of patients with SLE [4, 26]
RA	Used mainly in combination therapies (rarely in monotherapy) Used in monotherapy of RA can be used in case of intolerance of other DMARDs if low disease activity lasts no longer than 24 months and in case of the absence of bad prognostic factors	have an antagonistic effect on TLRs and thus inhibit the immune response [6] Interfere with antigen presentation and lysosomal acidification [30] Inhibit the production of RF antibodies, collagenase, and proteases which directly cause cartilage breakdown) (reviewed [6] Inhibit phospholipase A2 [6, 31] All effects motioned above partially explain the immunomodulatory impact of HCQ upon proinflammatory cytokines, such as IL-6, IL-1β, and TNF-α [32]	Block UV light in cutaneous reactions [6, 31] In advance or rapidly progressive disease should be used in combination therapy with other DMARDs (e.g., MTX or leflunomide) [33]
Primary Sjögren’s syndrome (pSS)	HCQ is recommended as first-line therapy for inflammatory musculoskeletal pain associated with pSS (recommendation of moderate strengths according to Sjögren’s Syndrome Foundation Clinical Practice Guidelines) [34]	↓ Tear fluid B-cell activator factor (BAFF) levels [35, 36] ↓ Disease activity and ↑ salivary flow [35] Improve the course of the disease (Valim) (Kruize)	HCQ improves fatigue, arthralgia, and myalgia [7, 37] ↓ Symptoms of dry eyes and ocular pain, ↑ cornea integrity (Valim) (Kruize) Reduce cardiovascular risk by decreasing dyslipidemia and hyperglycemia complications [17] are useful in the treatment of pSS, mainly in musculoskeletal pain and moderate disease activity [34–36] some studies suggest that HCQ has no significant effect in pSS as compared with placebo (randomized controlled trials) [38, 39]
Sarcoidosis	A second-line drugs Used in cutaneous sarcoidosis in case of GCS ineffectiveness applying locally Used in GCS treatment of cutaneous sarcoidosis after the failure of systemic GCS a beneficial effect in deforming skin lesions [40]	Particularly useful for cutaneous Decrease proinflammatory cytokine secretion [40] Inhibit antigen-presenting cells to CD4^+^ lymphocytes = > ↓ antigen processing and antigen presentation to the MHC II system = > ↓ decreased granulomatous lesions by T lymphocytes [41]	sarcoidosis (used in monotherapy or combination with GCS) and reduce the induction of new sarcoid skin lesions [41, 42] Cause involution of lung lesions (pulmonary sarcoidosis) [43] Used in moderate cutaneous sarcoidosis [8] Efficient in mild sarcoidosis-induced hypercalcemia and hypercalciuria (the best effect is achieved in combination therapy of GCS) [44, 45] Reveal efficacy in sarcoidosis-related arthritis [46, 47] Helpful in mild cranial neuropathies [48] Enable to taper or even discontinue GCS over several months [8, 46, 47] Cessation of AMDs cause relapse of sarcoidosis [8]
PM and DM	second-line drugs (after GCS) mainly used in DM to decrease skin lesions: HDQ sulfate monotherapy (2 × 200 mg/d) usually in combination therapy with GCS or quinacrine hydrochloride (1 × 100 mg/d)	Inhibit the activity of phospholipase A2, NK, IL-2, and TNF-α Decrease the phagocytic and chemotactic activity of immune cells Inhibit the formation of immune complexes Stabilize DNA Has an antioxidant effect [49]	CQ and HCQ reveal anti-inflammatory and immunosuppressive effects and improves cutaneous lesions of DM [49, 50] Are used mainly as an adjuvant to GCS or quinacrine therapy of patients with DM cutaneous lesions (rarely used in monotherapy) [49–51]. HCQ reduces skin lesions in juvenile DM [51]

Abbreviations: *APS*, antiphospholipid syndrome; *DM*, dermatomyositis; *DMARDs*, disease-modifying antirheumatic drugs; *PM*, polymyositis; *GCS*, glucocorticosteroids; *IL*, interleukin; *TNF*, tumor necrosis factor; *CQ*, chloroquine; *HCQ*, hydroxychloroquine; *MHC*, major histocompatibility complex; *MTX*, methotrexate; *TLRs*, Toll-like receptors; *RA*, rheumatoid arthritis; *RF*, rheumatoid factor; *NK*, natural killers; *SCLE*, severe cutaneous lupus erythematosus; *DLE*, discoid lupus erythematosus; *LEP* lupus erythematosus panniculitis

### AMDs in SLE

SLE is a systemic autoimmune disease, which is characterized by the inflammation of microvasculature leading to the involvement of multiple organs (causing lesions of the skin and mucus membrane, arthritis, neurologic disorder, kidney disease, and hematologic changes) [52]. The production of various auto-antibodies is a significantly important process in the pathogenesis of SLE, from which anti-dsDNA antibody and anti-Sm antibody are essential biomarkers [53]. The primary treatment and management of SLE include glucocorticoids (GCS) and HCQ, as well as nonsteroidal anti-inflammatory drugs, immunosuppressive agents, and biologic therapy [54].

**Fig. 1:**
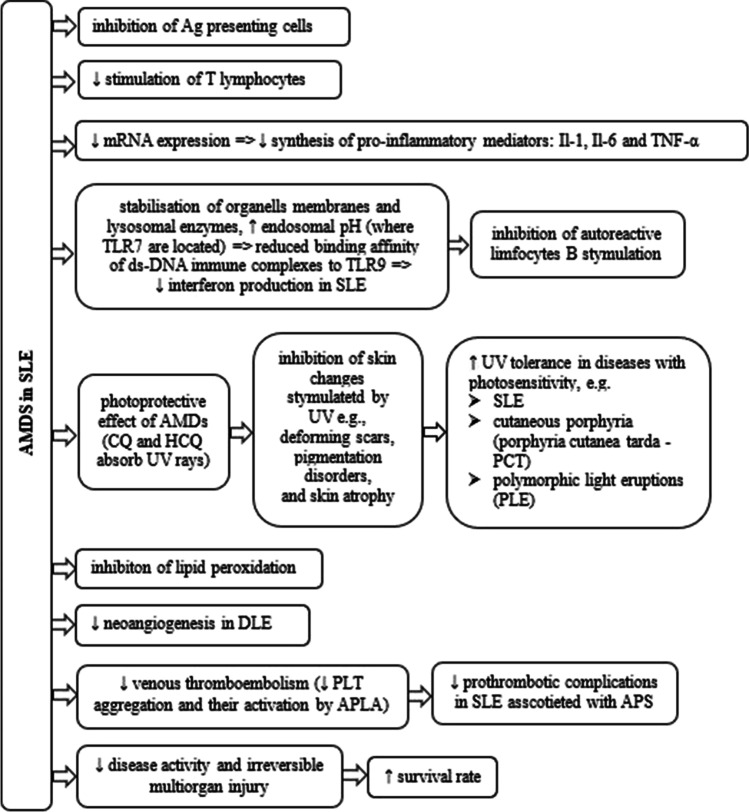
Beneficial effects of AMDs in SLE (DLE, discoid lupus erythematosus; APLA, antiphospholipid antibodies)

Previous studies have confirmed the efficacy of AMDs in SLE. AMDs demonstrate anti-inflammatory activity (inhibit the synthesis of proinflammatory cytokines) and antioxidant properties (Fig. 1). Recent data show a prominent role of CQ in the treatment of SLE. Along with glucocorticosteroids, CQ is recommended as the first-line treatment for SLE [55]. It alters the pH of lysosomes, where toll-like receptors (TLR)-7 and TLR9 are located. Thereby, it reduces the binding affinity of ds-DNA immune complexes to TLR9 and downregulates the production of interferon in SLE [24].

AMDs show good efficacy mainly in skin diseases, which are associated with joint and muscle inflammation [4]. However, CQ and HCQ are also used in more severe forms of SLE. AMDs decrease the activity of SLE by more than 50%, which helps to lower the doses of GCS [4]. Moreover, they protect against irreversible damage of organs, such as kidney and nervous system, polyserositis and cytopenia, thrombosis development, and loss in bone mass [4, 25, 56]. In patients with SLE associated with antiphospholipid syndrome (APS), which causes severe prothrombotic complications, AMDs decrease the risk of venous thromboembolism (by inhibiting the platelet aggregation and activating the antiphospholipid antibodies) [26]. Thus, they improve patient survival by reducing disease activity and preventing irreversible multiorgan injury [56].

Preclinical studies have shown that HCQ delays the onset of SLE by about 1 year. Patients treated with HCQ had a lower rate of autoantibody accumulation and a decreased number of autoantibody specificities after diagnosis. AMDs postpone the development of flare in the course of SLE [4, 27]. Moreover, a landmark trial showed that discontinuation of HCQ led to a nearly threefold higher risk of lupus exacerbation [57]. It was reported that a low level of serum HCQ in patients with SLE is associated with high disease activity and is considered a strong predictor of flare [58]. Thus, the use of AMDs is characterized by an increase in the long-term survival of patients with SLE [4]. From our clinical practice, AMDs should be continued, both in remission and in a flare.

The beneficial effect of AMDs is based on their reduction of cardiovascular risk factors, such as hyperlipidemia and diabetes mellitus. These medications have a positive influence on insulin resistance among patients with SLE [5]. In pregnancy, AMDs, mainly HCQ, decrease lupus activity and do not have a harmful effect on the fetus [4]. Moreover, they lower the risk of complications during pregnancy [29].

### AMDs in RA

RA is characterized by symmetrical inflammatory polyarthritis, often complicated by extra-articular manifestations and an increased rate of morbidity and mortality from CVDs [2, 59]. It affects ~ 0.5 to 1% of the overall population, and it damages cartilage and bone tissue leading to disability and reduced quality of life of the patient [2, 59, 60]. Early treatment of RA is associated with improved outcomes [61]. Immunosuppressive agents, such as MTX and LF, are frequently used in RA; nevertheless, AMDs, sulfasalazine, cyclosporine, and biologic agents are also implemented during the treatment of RA, usually as additive therapy.

AMDs are useful in the treatment of connective tissue disorders that are characterized by palindromic rheumatism [62, 63]. CQ and HCQ also inhibit lysosomal antigen degradation and prevent the activation of autoreactive T cells and subsequent inflammatory responses [30]. HCQ in RA decreases the number of tenders, swollen, and painful joints [33]. But it did not inhibit the radiographic progression [64]. Therefore, they are not used as first-line medications in RA because they do not reduce structural damage sufficiently, especially in comparison with other disease-modifying antirheumatic drugs (DMARDs) [65]. Compared to MTX or LF, AMDs show less activity, and they require a long time to show beneficial clinical improvement. Nevertheless, AMDs can combine with rheumatoid factors. Therefore, they are frequently used as a part of combination therapy [66], or in patients who have very mild disease, or in patients who show contraindications to other medications, such as MTX and LF [33].

### AMDs in primary Sjögren’s syndrome (pSS)

AMDs are also used in the treatment of pSS, which is an autoimmune exocrinopathic disorder characterized by lymphocytic infiltration of exocrine glands (mainly salivary and lachrymal glands) [67]. Such lymphoid infiltrations lead to dryness of the eyes and the mouth, as well as other surfaces connected to exocrine glands [68]. In addition to glandular involvement, systemic pathology has also been reported, such as respiratory or neurological disorders [67, 68]. It is noteworthy that pSS is associated with an increased risk of cancer, mainly non-Hodgkin lymphoma [69]. The mainstay of the treatment for the sicca symptoms is local therapy (saliva substitutes and artificial tears) and that for the milder systemic symptoms is HCQ [7]. The treatment of systemic symptoms (extra-glandular manifestations) includes GCS, AMDs, immunosuppressive drugs, and biologic agents [70].

Various studies have shown that HCQ is useful in the treatment of pSS, and it alleviates the signs and symptoms of dry eyes [35, 36]. AMDs show the best effect in the initial stage of the disease without any severe damage to the organ (“burned out” phase), and they improve the course of the illness (decrease fatigue, joint pain, erythrocyte sedimentation rate (ESR), rheumatoid factor (RF) level, gamma globulin level and diminish glandular involvement) [37]. Moreover, they inhibit salivary and serum B-cell activator factor (BAFF) [35, 36]. This helps to reduce disease activity and increase salivary flow [35].

However, some studies do not confirm the efficacy of HCQ in pSS. A randomized controlled trial showed that after 24 weeks of treatment with HCQ, there was no significant improvement in the symptoms of pSS compared with placebo [38]. There was no apparent clinical benefit in the case of dry eyes and systemic inflammation in pSS when HCQ was administered at a dosage of 300 mg daily for 12 weeks [39]. Nevertheless, AMDs have an anti-inflammatory, immunosuppressive, and immunomodulatory role, and they are recommended as first-line treatment for inflammatory musculoskeletal pain associated with pSS (recommendation according to Sjögren’s Syndrome Foundation Clinical Practice Guidelines) [34]. In clinical practice, they are mainly used to treat mild disease without the involvement of organs (e.g., lung fibrosis or severe neuropathy). If an organ is involved, then immunosuppressive medications are required (e.g., cyclophosphamide in pulmonary fibrosis) [34].

### AMDs in polymyositis (PM) and dermatomyositis (DM)

Idiopathic inflammatory myopathies (IIM) encompass a heterogeneous group of connective tissue disorders that are characterized by chronic inflammation of striated muscle. The symmetrical weakness of proximal muscle groups, decreased muscle endurance, and chronic inflammation in muscle tissue are the predominant symptoms of IIM [71]. The most common subsets of IIM include adult PM, adult and juvenile DM, immune-mediated necrotizing myopathy, and sporadic inclusion body myositis (IBM) [72]. PM is characterized by symmetrical proximal and progressive weakness in the muscles of limbs, whereas in DM, muscular disorders co-exist with skin abnormalities, e.g., heliotrope rash and Gottron’s papules [73]. Typical complaints include problems in walking, climbing stairs, or lifting an object above their head. Muscle weakness is associated with elevated blood levels of muscle enzymes, particularly creatine phosphokinase (CK). Both PM and DM are often related to nonmuscular manifestations such as interstitial lung disease (ILD), arthropathy, cardiomyopathy, and malignancies [74]. The diagnosis requires testing of auto-antibodies, histological evaluation of a skeletal muscle biopsy, and further tests, including muscle magnetic resonance imaging (MRI) and electromyography (EMG) [75]. The treatment of PM/DM includes GCS and immunosuppressants. AMDs are also used in the treatment of inflammatory myopathies, particularly the cutaneous symptoms of DM [49, 76].

Various case reports or clinical case series have shown beneficial effects of AMDs. According to these reports, AMDs can be administered alone or in combination with GCS [49–51]. HCQ is usually used in combination with GCS and is good to treat skin conditions of patients with DM. It can completely resolve the lesions on the skin and may enable GCS tapering [50]. The efficacy of AMDs has been demonstrated in adults [50] and childhood DM [51]. Some studies highlight the positive effect of AMDs in the treatment of cutaneous lesions in dermatomyositis [50]. However, they may also cause mild-to-moderately severe vacuolar myopathy and may be responsible for severe and death-leading cases with ventilatory failure [77]. Moreover, an increased risk of Herpes zoster infection has been described in patients with PM/DM [78]. In addition to these casuistic descriptions, AMDs have shown excellent safety profiles, but their use in inflammatory myopathies should be carefully monitored in clinical practice, which can be achieved by analyzing patient symptoms and measurement of muscle enzymes [3, 15, 79].

### AMDs used in sarcoidosis

Sarcoidosis is a multisystem granulomatous disease characterized by the presence of noncaseating granulomas [80]. The course of this disease can be self-remitting within 12 to 36 months (in more than 60% of the patients) or can become chronic, requiring prolonged treatment (in approximately 10% to 30% of the patients) [81]. In the case of patients in whom sarcoidosis does not cause any significant symptoms or organ dysfunction, the treatment is not compulsory. Systemic therapy is required in life-threatening conditions with organ involvement such as advanced pulmonary fibrosis or pulmonary hypertension, central nervous system disorders, heart, or renal sarcoidosis [82]. If organ damage is observed, the first-line treatment for sarcoidosis is GCS, which reveals therapeutic effects. In rare cases, the disease fails to respond to GCS monotherapy or combined GCS and second-line immunosuppressants. Combined therapy is mainly used to achieve disease control or GCS sparing, mostly in cases, when multiple side effects are present [43, 82].

When treatment is indicated, oral corticosteroids are usually recommended because they show good efficacy in a short period. AMDs are recommended mainly in cutaneous sarcoidosis, and they cause the regression of granulomatous changes on the skin (the minimum doses of CQ and HCQ have the same effect as the maximum doses) [42]. AMDs are suspected to impair the release of cytokines and weaken the process of antigen presentation by monocytes, macrophages, and dendritic cells to CD4-positive helper T cells [40]. Moreover, CQ has demonstrated a positive effect in the treatment of symptomatic persistent pulmonary sarcoidosis, but the data are quite limited [43]. In sarcoidosis, AMDs are used in patients who fail to respond to GCS treatment or show contraindication to GCS. Since sarcoidosis is often a chronic condition, long-term treatment with GCS may cause significant toxicity. Therefore, GCS-sparing agents are often indicated in patients requiring prolonged therapy [82]. Therefore, AMDs are mainly recommended in cutaneous forms of sarcoidosis, usually along with rapidly working GCS, whose doses could be slowly decreased.

## Mechanism of antiviral action of AMDs

AMDs demonstrate broad-spectrum antiviral effects (Table 2). For example, more than 20 years ago, CQ was shown to inhibit HIV-dependent replication [83]. Other examples include inhibition of RNA viruses such as hepatitis A or C; influenza A, B, and H5N1 viruses A; poliovirus; rabies; dengue; or Ebola, as well as DNA viruses (hepatitis B and herpes simplex virus) [84]. Moreover, the positive effect of AMDs has been reported in the treatment of different types of coronaviruses such as CoV-229E (in vitro studies) [85], HCoV-OC43 (studies on animal models) [86, 87], and SARS-CoV-1 [11, 88].

**Table 2:** The activity of antimalarial drugs (AMDs) in various viral diseases

Type of virus	Drug characteristics
The (hydroxy) chloroquine activity in viral infection	
HIV	CQ/HCQ increases endosomal pH CQ impairs posttranslational protein modification of viral protein and decreases glycosylation of the gp120 envelope glycoprotein; in consequence, the synthesized viral cells are not infectious [89] ^⟡^ HCQ (800 mg/d for 8 weeks) administration in asymptomatic patients reduces viremia in plasma, preserves CD4^+^ T cell counts and proliferative responses, and lower serum IL-6 concentrations [90] **
HSV	CQ inhibits the budding process in the HSV model and the newly synthesized viral HSV-1 particles do not have infectious properties [91] ^⟡^
Dengue-2 virus	CQ changes endosomal pH and impairs the viral maturation by affecting the standard proteolytic processing of the flavivirus protein to M protein [92] ^⟡^ Inhibits IFN-α, IFN-β, IFN-γ, TNF-α, IL-6, and IL-12 gene expression in U937 cells infected with dengue-2 virus [93]^⟡^ Cause an increase in pH cytosol and enables endocytosis; as a result virus replication is deturbed [11] **
Influenza H5N1	CQ is a potential broad-spectrum antiviral drug used for influenza H5N1 in an animal model [94].
The activity of CQ in various coronavirus infection	
Coronavirus HCoV-229 (alfa-coronavirus)	Under in vitro conditions, CQ hinders the activation of the p38 MAPK (MAPK activity is necessary for virus replications) and thus, inhibits the replication of HCoV-229E in epithelial lung cell cultures [85] ^⟡^
Coronavirus HCoV-OC43 (beta-coronavirus 2a)	HCQ inhibits quinone reductase 2, which is involved in the biosynthesis of sialic acid, which builds viral receptors [86] ^⟡^ ↓ Lethal infections of newborn mice with the HCoV-O43 when administered through the mother’s milk [87] ^○^
MERS-CoV	CQ inhibits M proteins accumulation in the Golgi complex beyond the site of virion budding [95] ^⟡^
SARS-CoV-1	Interferes with ACE2 receptor glycosylation and prevents binding of the virus to target cells [96] ^⟡^ Prevents the attachment of viral proteins to endosomal membranes by increasing pH of endosomes and thus, does not allow the viral genome to be released into the cytosol (replication cannot be initiated) [88] ^⟡^
SARS-CoV-2	AMDs as weak bases accumulate in the acidic environment of endolysosomes and other acidic cell organelles and alkalize endosomes [97] ^⟡^ Interfere with the terminal glycosylation of ACE2 and affect virus binding [97]^⟡^ **Clinical studies:** Improve pneumonia symptoms, laboratory tests, and decrease the progression to severe or critical conditions [98] lower mortality risk (n = 2541, HCQ 2 × 400 mg first day, then 2 × 200 mg on days 2–5 with/or without azithromycin) [99] * lower risk of hospital discharge of patients given treatment up to 28 days (HCQ 2 × 800 mg in 6 h, then 2 × 400 mg at 12 h a first day, then 2 × 400 mg/day on days 2–10, or until discharge); no significant difference in mortality risk between HCQ treated patients and control group, but higher risk of symptoms exacerbation in patients treated HCQ, who required invasive mechanical ventilation [100]* no significant differences between groups in conversion to negative SARS-CoV-2 RT-PCR and the degree of symptom recession after 4 weeks (n = 150, HCQ 1200 mg/day for 3 days (loading dose), then 800 mg/day for 2 weeks if mild/moderate, or 3 weeks if severe) significantly more adverse effects in the HCQ arm (mainly no severe: diarrhea, blurred vision, no cardiac arrhythmic events) [101]* increase mortality from any cause in the HCQ group than the non-HCQ group (n = 368, unspecified dose of HCQ azithromycin males over 65 years old, predominantly African American veterans exhibiting high rates of hospitalization), but no significant difference in ventilation risk in either treatment group compared to the control [102]* HCQ (800 mg first day, then 400 mg on days 2–7) has no significant influence on the prevention of SARS-CoV-2 transmission and caused a higher incidence of no-serious adverse events in the treatment group (n = 2314) [103]*

Abbreviations: *ACE2*, angiotensin-converting enzymes 2; *MAPK*, mitogen-activated protein kinase; *IFN*, interferon; *TNF*, tumor necrosis factor; *IL*, interleukin; *HIV*, human immunodeficiency syndrome; *HSV*, herpes simplex virus; *MERS*, the Middle East respiratory syndrome coronavirus; *SARS-CoV-1*, severe acute respiratory syndrome coronavirus 1; *SARS-CoV-2*, severe acute respiratory syndrome coronavirus 2

^**^Adequately power human study (a randomized, double-blind, placebo-controlled study)

^*^Inadequately power (lack of placebo and/or lack of randomization)

^○^Animal study

^⟡^In vitro study

Initially, the suspected mechanism of action of CQ against SARS-CoV-2 was based on its activity on other viruses, mainly SARS-CoV-1, dengue-2 virus, and influenza A H5N1 virus [11, 15, 49, 94]. In viral infections, AMDs can impair the replication of some viruses by reducing the efficiency of endosome-mediated virus entry [15, 94]. These drugs also decrease the activity of low-pH–dependent proteases in trans-Golgi vesicles [15]. It was shown that CQ inhibits the initial step of the viral cycle by interfering with the binding of viral particles to the cell surface receptor. It inhibits quinone reductase 2, which participates in the biosynthesis of sialic acids [104], which are part of the transmembrane proteins and are involved in the recognition of ligands. Many coronaviruses (e.g., HCoV-O43) use sialic acid residues as receptors [86]. Thus, these data gave the background to suspect that AMDs might be active in SARS-CoV-2 infection.

## AMDs in SARS-CoV-2 infection

Disease caused by the new beta-coronavirus SARS-CoV-2 was first reported in the Chinese city of Wuhan, but the rapidly spreading infection with life-threatening complications caused the WHO to declare a global pandemic. This virus is similar in structure to the SARS-CoV-1, and it contains 80% of the same nucleotides. The disease caused by SARS-CoV-2 is called COVID-19 (coronavirus disease 2019) [105]. Since the epidemic started, the entire medical world began to look for new and effective drugs, which will help to control the spread of the disease. Because CQ and HCQ have been reported to have broad-spectrum antiviral activity, they have been considered for use in the treatment of COVID-19 [106].

**Fig. 2:**
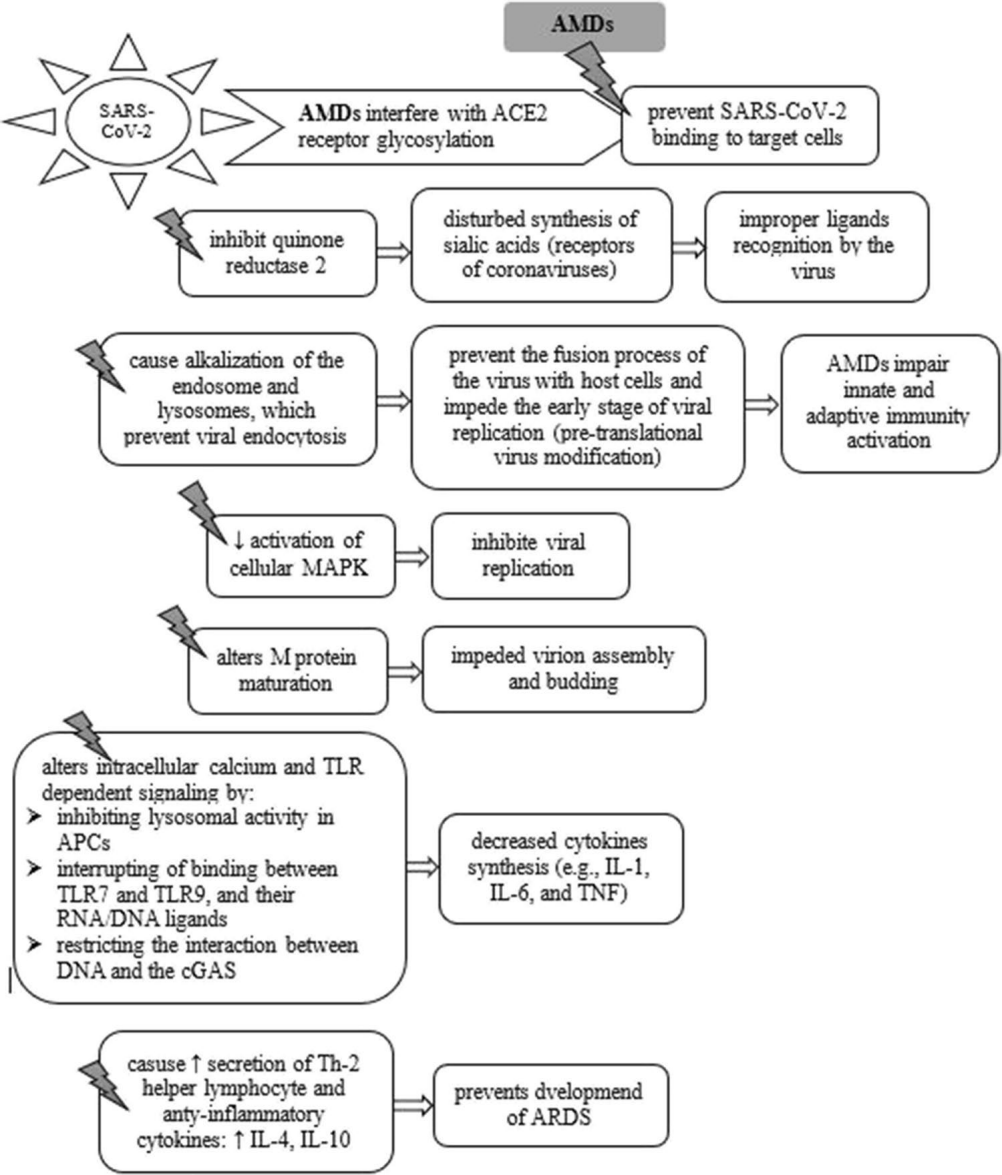
The possible model of chloroquine activity in novel severe acute respiratory syndrome coronavirus 2 (SARS-CoV-2) infection (APCs, antigen-presenting cells; TLR, toll-like receptors; cGAS, cyclic GMP-AMP synthase; MAPK, mitogen-activated protein kinase)

The exact antiviral mechanisms of AMDs are not known, but many mechanisms of action have been suggested (Fig. 2). The effect of these drugs in inhibiting SARS-CoV-2 is based on the fact that CQ influences both entry and intracellular stages of the SARS-CoV-2 replication in Vero E6 cells [107]. Many coronaviruses bind to sialic acid and this process is required for viral replication. It has been proved that CQ decreases the biosynthesis of sialic acid. Therefore, it is suspected that SARS-CoV-2 similarly binds to sialic acid, and this binding can be inhibited by CQ [108].

Another mechanism, which is involved in the activity of CQ is its interaction with the surface receptor of the angiotensin-2 converting enzyme (ACE2). S protein of the virus attaches to host receptor ACE2. Similar to SARS-CoV-1, SARS-CoV-2 virus also uses the same ACE2 receptor. These receptors are present on the surface of the lung, heart, kidneys, and intestines [96, 109]. Recent data shows that SARS-CoV-2 binds to ACE2 expressed on pneumocytes [109]. CQ reduces the ACE2 receptor terminal glycosylation on the surface of cells and interferes with the binding of SARS-CoV-2 to the ACE2 receptor [97, 110].

The penetration of coronavirus also occurs through the endosomal pathway [111]. AMDs increase the endosomal pH of the cell, which affects the binding of the virus to the cell and interferes with the glycosylation of the cell surface receptors [15, 94]. An increase in the endosomal pH by CQ inhibits the formation of cathepsins, which require an acidic environment for the optimal cleavage of SARS-CoV-2 spike protein [112]. Thus, CQ, by modulating the acidification of endosomes, prevents virus/cell fusion with host cells and subsequent viral replication [97, 107]. In in vitro studies, CQ inhibits the replication of SARS-CoV-2 at a low-micromolar concentration (EC_50_ = 1.13 μM in Vero E6 cells) [107].

An additional mechanism of action of CQ, which is suspected in COVID-19, includes the inhibition of MAPK kinases, inhibition of proteolysis of the M protein and alteration of virion assembly, and budding. CQ might indirectly decrease the production of proinflammatory mediators and/or by activating anti-SARS-CoV-2 CD8^+^ T cells and the inhibition of production of cytokines such as IL-1, IL-6, and TNF-α [113]. The inhibition of the production of proinflammatory mediators may reduce the effects of a “cytokine storm.” Excess cytokine synthesis results from the Th-2-dependent response during the course of coronavirus infection, which leads to the development of various symptoms [14, 15]. Thus, decreasing the excess production of proinflammatory markers can deteriorate the severity of the viral infection. Moreover, during the initial phase of SARS-CoV-2 infection, AMDs increase the secretion of Th-2 helper lymphocyte cytokines (IL-4 and IL-10) [114]. In addition, CQ prevents exacerbated activation of Th-1 lymphocytes, which are responsible for the development of acute respiratory distress syndrome (ARDS) [15].

In addition to inhibiting the entry of viruses, CQ inhibits the secretion of IL-2, which is involved in the differentiation of T-2 cells into the Th-2 subset [115]. As a result, the proliferation of Th-2 lymphocytes is reduced. Thus, it is hypothesized that CQ and HCQ impair the immune response to SARS-CoV-2 infection and prevent the progressive course of the disease [114]. In summary, AMDs in coronavirus infection interfere with ACE2 to block the invasion of the virus, increase endosomal pH required for the fusion of the virus, and show mild immune suppression.

## AMDs in clinical practice

On 13 March 2020, the Office of Registration of Medicinal Products, Medical Devices, and Biocidal Products in Warsaw (Poland) issued a new therapeutic indication for medicines containing CQ, consisting of the addition of “supportive treatment in beta-coronavirus infections such as SARS-CoV-1, MERS-CoV, and SARS-CoV-2.” This drug was used to treat patients from the Wuhan region and several other cities in China. Initially, CQ and HCQ were approved for the treatment of COVID-19 based on their in vitro antiviral activity [13] and antiviral properties against other viral infections, e.g., HIV [83] and SARS-CoV-1 [97, 116]. Given these facts, the first clinical trials tried to check an inhibitory effect of AMDs on viremia [15, 16]. In rheumatic diseases, AMDs reveal their full anti-inflammatory and immune regulatory activity within a few weeks (about 1–3 months), but their antiviral effect is quite rapid [17].

The preliminary observation revealed that CQ inhibits the progression of clinical and radiological signs of pneumonia, facilitates the elimination of the virus up to the stage of indeterminate viremia, shortens the duration of the disease, reduces hospitalization time, and has no severe side effects [16]. HCQ used in COVID-19 for 3 days enhanced virus clearance, and azithromycin reinforced the antiviral efficacy [17].

## CQ versus HCQ

Because CQ and HCQ (a derivative of chloroquine) have similar chemical structures and mechanisms of action, they are equally used in the treatment of viral infection. Both AMDs are active against SARS-CoV-2 under in vitro studies; however, HCQ seems to differ from CQ in a few aspects [13, 107]. In vitro [13] and in vivo study has confirmed [98] the effect of HCQ in COVID-19 infection. But HCQ was more potent than CQ in inhibiting SARS-CoV-2 infection. The therapeutic effect of HCQ was confirmed by a randomized controlled clinical trial (ChiCTR2000029559). This study revealed that patients on HCQ therapy had significantly shortened clinical recovery time, quick resolution of symptoms (such as fever and cough), and more than 80% of patients had improved pneumonia compared to the control group without HCQ therapy [98]. HCQ demonstrated substantially fewer side effects [117] and seems to show a more potent antiviral activity than that of CQ [13, 107]. Therefore, HCQ was recommended in SARS-CoV-2 treatment in an expert consensus statement from Shanghai [118]. Moreover, it does not cause multiple drug–drug interactions than that of CQ [13].

Based on an in vitro study [107], HCQ was rapidly introduced into clinical use, and preliminary reports suggested an improved viral clearance and clinical outcomes in patients with COVID-19 receiving a 10-day course of HCQ [16]. A small French pilot study, randomizing 36 patients with COVID-19, suggested accelerated viral clearance in patients treated with a combination of HCQ and azithromycin [17]. Moreover, HCQ equally as CQ influences macrophage activation and cytokine storm [119]. This results in the decreased production of proinflammatory mediators such as IL-1, IL-6, and prostaglandins [120]. Moreover, HCQ has antithrombotic effects, which prevents the formation of micro-thrombus during endothelial damage caused by SARS-CoV-2 infection [121, 122].

The earliest use of AMDs has shown that HCQ shows a positive effect in the treatment of COVID-19 when given in combination with azithromycin leading to a complete and rapid viral clearance (clinical trials) [17]. However, some studies did not confirm this hypothesis. Magagnoli et al. conducted a retrospective study and analyzed the use of HCQ with and without azithromycin and with the control group (without HCQ) [102]. They observed that the risk of death from any cause was higher in the HCQ group (adjusted HR = 2.61; 95% CI: 1.10–6.17; p = 0.03) than that of the non-HCQ group (the authors analyzed a cohort of 368 males, predominantly African American veterans). Moreover, the risk of necessary ventilation or the risk of death after ventilation was also not significantly different among the three groups [102]. Similar doses of HCQ and azithromycin were used in the prospective case series on 11 patients with severe infection with SARS-CoV-2, in which viral clearance and clinical outcome were not improved by this combination. Molina et al. [123] conducted a study on 181 patients with severe infection with SARS-CoV-2 showed that HCQ did not significantly reduce ICU admission, death on the seventh day after hospitalization, or reduce the incidence of ARDS compared to those who did not receive HCQ [124].

## The dose of CQ and HCQ in COVID-19 treatment

In vitro study confirmed the inhibitory effect of CQ against SARS-CoV-2 at concentrations that is much lower than cytotoxic level [107]. An excellent safety profile and antiviral activity allowed to consider CQ in the treatment of COVID-19. In a study conducted on 13 severe to critically ill patients with COVID-19 admitted in the ICU, the authors recommended the use of HCQ 800 mg once on the first day to rapidly achieve therapeutic levels, followed by 200 mg twice daily for 7 days [125]. A similar dosage was recommended by Yao et al. for HCQ; they administered 400 mg bid on the first day and the maintenance dose of 200 mg bid for 4 days [13]. The guidelines of SIMIT Lombardy section included CQ/HCQ in the treatment of severe to critically ill patients with COVID-19 [126].

## Are AMDs always effective in viral infections?

Some studies suggest limited benefit from CQ/HCQ in the treatment of COVID-19 in general. There is evidence contradicting the use of these drugs in viral infections, underlying their adverse effect on viremia, which is explained by the drug-mediated delay of the adaptive immune response to infection. This suspect is evidenced by studies regarding the treatment of the chikungunya virus by AMDs in animals [127]. In the case of chikungunya infection, CQ administered as a preventive therapy increased the severity of symptoms and delayed the elimination of the virus in monkeys (probably by inhibiting the specific cellular response) [9]. Moreover, CQ increased the risk of late complications such as joint infection one year after the disease onset; however, when compared to placebo, there were no significant effects on the acute phase of fever [128]. Similarly, in humans, the adverse effects of CQ have been recorded during the treatment of acute-phase chikungunya fever [128].

CQ was ineffective in the prevention of influenza in humans [10] and the treatment of dengue [11]. It was also ineffective in eliminating SARS-CoV-1 infection in mice [14]. It may increase symptom severity and mortality in viral diseases, such as encephalomyocarditis, and it increases viral titers in various organs [129]. CQ might predispose patients with DM/PM to developing herpes zoster, particularly in women and patients with DM, independent of disease status, therapy, and demographic features [78].

A recent study analyzed the CQ regiment and showed that a high dose (600 mg CQ twice daily for 10 days or total dose of 12 g) is not sufficiently safe because it increased the QTc value to more than 500 ms (25%), and the trend toward higher mortality. Therefore, it should no longer be used in patients with a severe infection of SARS-CoV-2 [130].

Recent studies include larger groups of SARS-CoV-2 infected patients using AMDs, mainly HCQ. The study of Tang et al. analyzed 150 patients with mild COVID-19 who were treated with standard regimens with or without HCQ (ChiCTR2000029868). This study showed no significant differences between groups in conversion to negative SARS-CoV-2 RT-PCR and the degree of symptom resolution after 4 weeks. Moreover, the authors observed significantly more side effects in the HCQ arm (mainly no severe). Beneficial was the earlier normalization of CRP in the patients using HCQ with standard care, but the differences were not significant [101]. Another study analyzing adult outpatients (n = 423) investigated the efficacy of HCQ in the reduction of COVID-19 severity (March–May 2020, randomized, double-blind, placebo-controlled trial ClinicalTrials.gov: NCT04308668). The patients were treated with HCQ (first dose 800 mg once, followed by 600 mg in 6 to 8 h, then 600 mg daily for 4 more days) or placebo. After 2 weeks of such treatment, the change in symptom severity did not differ between the HCQ and placebo groups. Adverse effects of HCQ were observed in 43% of patients (92 of 212) versus 22% receiving placebo (46 of 211, p < 0.001). The authors concluded that HCQ did not substantially reduce symptom severity in outpatients with early, mild COVID-19 [131].

Recently two studies analyzing the use of antimalarial drugs with azithromycin were published. The study of Cavalcanti et al. who analyzed the effect of therapy in three-group receiving standard care, standard care plus HCQ (2 × 400 mg/day), or standard care plus azithromycin (500 mg/day for 7 days) with HCQ (2 × 400 mg/day) in mild-to-moderate COVID-19. This study showed that HCQ ± azithromycin did not improve clinical status at 15 days compared with standard care (ClinicalTrials.gov number, NCT04322123). Moreover, prolonging the corrected QT interval and elevation of liver-enzyme levels were more frequent in patients receiving HCQ with or without azithromycin compared to standard care without these medications [132]. Conversely, the trial published by Arshad et al. showed that COVID-19 patients (n = 2541) treated with HCQ or azithromycin or a combination of two showed beneficial effects such as a reduction in COVID-19 associated mortality (when controlling for COVID-19 risk factors) [99]. Shamshirian et al., in meta-analysis describing HCQ use in COVID-19 treatment, revealed no clinical benefits to patients receiving HCQ with standard care [133].

On April 2020, the US CDC published information stating, “hydroxychloroquine and chloroquine are under investigation in clinical trials” [134]. However, in June 2020, FDA annulled the emergency use of CQ and HCQ to treat patients with COVID-19, considering the adverse effects of these medications [135]. Thus, even if AMDs might reduce the symptoms and improve the course of SARS-CoV-2 infection, some studies show that they have no benefits [124, 136] or are even hazardous to health [102].

## Adverse effects of AMDs in rheumatic diseases

A high safety profile usually characterizes AMDs in rheumatic diseases; however, they are used in smaller doses (usually CQ 250–500 mg/day; HCQ 200–400 mg/day) than that of COVID-19 treatment [79]. In 250 mg/day, chloroquine provides a stable concentration in the range of 100–500 ng/ml. The onset of action of HCQ is observed after 4–6 weeks, but full efficacy can be assessed after 3 to 6 months [137, 138]. Nevertheless, of used dose, these medications can cause serious side effects, which should be monitored. The significant side effect of AMDs, mainly caused by CQ, is retinopathy. In prolonged and strict regimens, retinopathy depends on the cumulative dose [139]. Consequently, AMDs should be used in patients with no ocular problem, and the patients should be regularly examined by an ophthalmologist (a baseline eye exam and follow-up exam every 6–12 months are requisite) [140].

In addition to retinopathy, AMDs can cause other severe complications such as cardiac toxicity. Every patient should be strictly monitored and the therapy should be immediately ceased if there are changes in their electrocardiogram, such as prolonged QT interval, which is the risk of arrhythmia [123]. Other rare side effects of AMDs include nausea, agranulocytosis, and hemolysis in patients with the deficiency of glucose-6-phosphate [79]. Among the two available antimalarial drugs, HCQ is the one with the lowest retinal toxicity [4, 140], but CQ appears to be more effective [79].

The use of AMDs in smaller doses in rheumatic diseases is very safe, and severe complications such as antimalarial myopathy, cardiomyopathy, or maculopathy are rarely observed [141]. These medications are usually administered in smaller dosages for an extended period (not higher than 500 mg of HCQ per day and not more than 400 mg of CQ per day, usually 250 mg CQ or 200 mg HCQ is administered as a single evening dose). As a result, they show a high safety profile and are very well tolerated. Thus, in rheumatic diseases, toxicity related to AMDs is infrequent, mild, and usually reversible, with HCQ having a safer profile than that of CQ [4].

## Adverse effects of AMDs in COVID-19 treatment

There are high differences between the dosage of AMDs in rheumatic and viral diseases. In SARS-CoV-2 infection, AMDs are used at a high dose to give the effect of drug saturation (600 mg CQ twice daily for 10 days (total dose 12 g)) [130]. This dose of CQ requires strict monitoring of the side effects, particularly in critically ill patients with renal and hepatic disorders (related to changed metabolic pathways and excretion of drug metabolites). Even AMDs are administered in adequate doses, and under close monitoring, their therapeutic window is narrow, which increases their risk of toxicity. Because AMDs have strong tissue tropism for the kidneys and liver, the risk of adverse effects will be higher in critically ill patients with renal or hepatic dysfunction than in patients with less severity [79].

AMDs can cause direct myocardial toxicity leading to cardiomyopathy and arrhythmias. They prolong the QT interval and increase the risk of arrhythmia [79]. Some studies did not confirm a significant effect of HCQ in patients with severe COVID-19 compared to patients without HCQ, but they show that 8 patients from 84 participants in the HCQ group (9.5%) discontinued HCQ after 4 days due to prolonged QT interval or first-degree atrioventricular block [124]. The same side effect was observed in a study of severe to critically ill patients with COVID-19 admitted in the ICU, who were administered with HCQ orally; however, it was withdrawn in 2 patients out of 13 participants [125]. The use of CQ with concurrent macrolides (azithromycin) and quinolone was not recommended as there is a risk of the prolonged QT interval [142]. Various studies have shown that HCQ does not reduce mortality or reduce the duration of the disease. This was particularly seen in a severely ill patient with advanced pneumonia [143]. In a previous study, the positive effect of AMDs has been described (mainly hypolipemic and hypoglycemic), but it also shows that they can cause hypoglycemia in severely ill patients [144].

Recent studies have shown that 30% of the patients with COVID-19 experience cardiac injury, which can further increase the risk of cardiomyopathy and arrhythmias. In such a situation, AMDs should be carefully used because the risk of cardiotoxic events is very high, particularly when they are used at a high dose [18, 19]. Because of the small difference in the therapeutic and toxic dose and life-threatening cardiovascular complications in the case of an overdose of CQ/HCQ, these drugs should only be used under strict medical supervision. In such cases, the choice of medication is crucial. HCQ has similar pharmacokinetics and mechanism of action as CQ but shows substantially fewer side effects [117, 145].

In June 2020, the US Food and Drug Administration (FDA) withdrew the conditional authorization for the use of AMDs in patients with emergency use in COVID-19 treated in hospitals unless they can participate in clinical trials. According to FDA, AMDs are unlikely to be effective in the treatment of patients with COVID-19 and that their benefits do not outweigh the risks identified in emergency use authorization (EUA), because of severe adverse events, mainly cardiological [146]. Moreover, the FDA issued a warning that AMDs reduce the antiviral activity of remdesivir. Because of the lack of clinical data, patients treated with the remdesivir for COVID-19 should not receive AMDs [147].

In conclusion, in the case of the prolonged QT interval, ocular problems, and neurological symptoms, AMDs should be withdrawn from the treatment of both rheumatic and viral diseases.

## Prevention of toxicity of AMDs

**Fig. 3:**
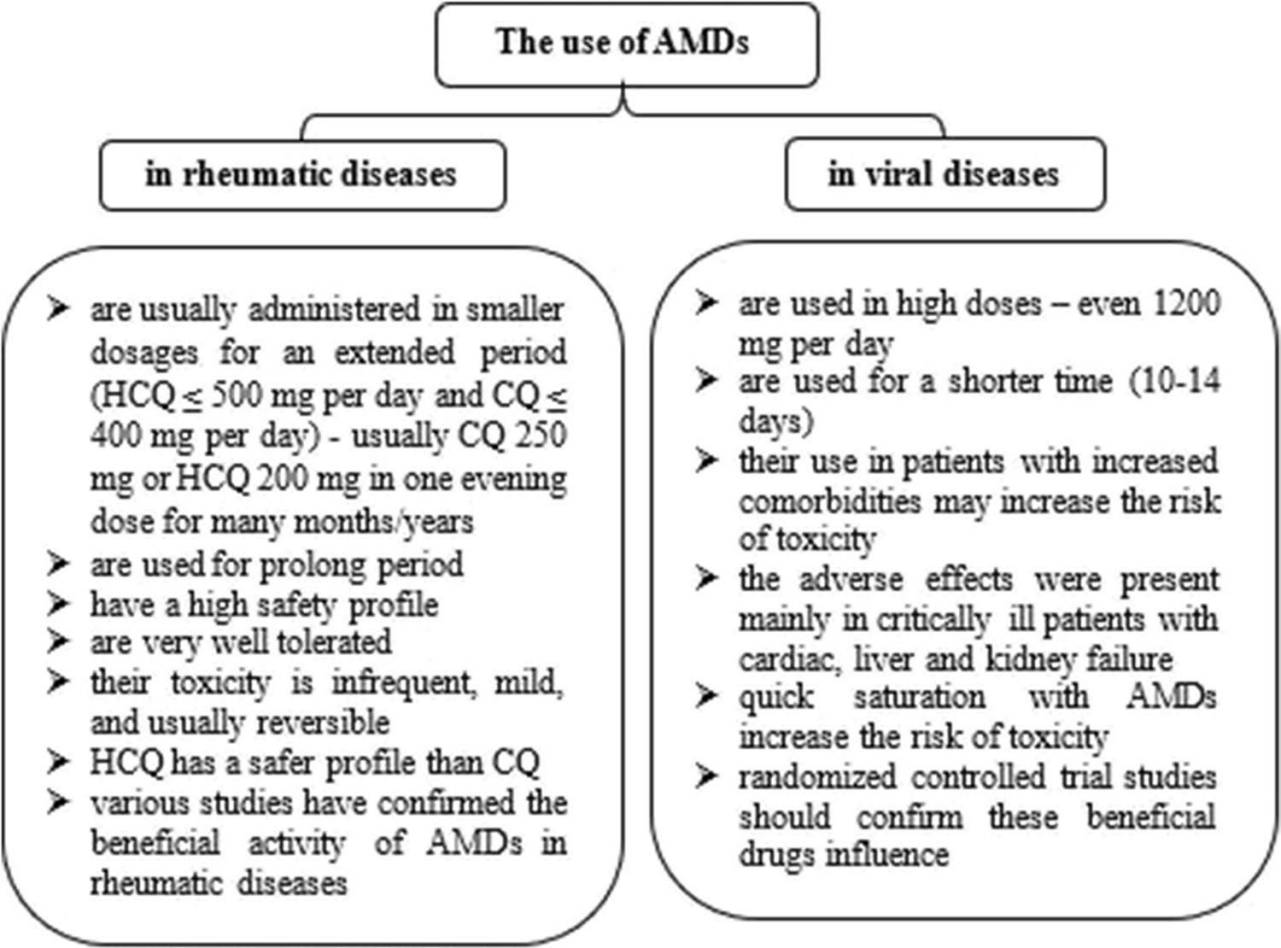
Comparison between the use of antimalarial drugs (AMDs) in rheumatic and viral diseases

Patients treated with AMDs at high doses and for a short time should be monitored for daily blood counts, electrolytes, and cardiac enzymes, as well as electrocardiogram monitoring [142] (Fig. 3). Before the start of the treatment, patients should be interviewed about visual changes during treatment; however, the American Academy of Ophthalmology does not recommend retinal screening before the short-term use of CQ [148]. In every case, the contraindications to HCQ therapy should be considered before ordering this medication (deficiency of glucose-6-phosphatase, QTc prolongation in the electrocardiogram, history of allergy to hydroxychloroquine, and the risk of retinopathy and cardiomyopathy) [17].

## Conclusion

AMDs show therapeutic effects in various diseases, and recently, the evidence for their potential benefit continue to grow in autoimmune pathologies. The use of AMDs in connective tissue disorders is well established, and they reveal many beneficial effects (improve cutaneous and musculoskeletal symptoms, decrease the activity of the disease and organ damage, and reduce cardiovascular risk). In recent decades, many studies have reported the wide-ranging benefits of CQ and HCQ. However, the dose of AMDs in rheumatic diseases is much lower than that in SARS-CoV-2, and the time to achieve drug saturation is longer. The full therapeutic effect of these medications can be seen later, but the risk of severe complications is low. In addition, antimalarial myopathy, cardiomyopathy, or maculopathy are rarely observed.

AMDs change the pH of the endosomes during viral replication and they inhibit “cytokine storm” (mainly TNF-α and IL-6). Given that, both CQ and HCQ have been considered attractive candidates for the potential treatment of COVID-19. Recent studies have revealed both the beneficial and harmful effects of AMDs. The discrepancies in the use of CQ and HCQ might be due to various patient populations, which were characterized by a different course of the disease (mild vs. severe), the various dosage of the drug (400–1200 mg), COVID-19 association with other chronic diseases (mainly CVDs and respiratory disorders), lack of control group, and the use of CQ/HCQ with other drugs. Thus, the recommendations for the use of AMDs should be confirmed by randomized controlled trials. In a viral infection, the effect of AMDs is quickly required, and the drug saturation can be achieved by high doses. In such circumstances, the possibility of side effects is higher than that when a slow saturation is achieved in rheumatic diseases. The safety profile and dosage regiment of CQ/HCQ should be mainly assessed in critically ill patients with detailed analysis of other risk factors which may influence the effectiveness of AMDs (e.g., old age, multiple comorbidities, the synergistic effect of drugs, or delay therapy of COVID-19).

Therefore, we conclude that AMDs are safe and should be considered in the treatment of both rheumatic diseases and mild viral diseases. Furthermore, results from the ongoing trials are required to determine the effectiveness and safety of AMDs in treating COVID-19. Considering the fact that CQ and HCQ have excellent efficiency and low toxicity, they should be considered candidate drugs for post-exposure prophylaxis of SARS-CoV-2. However, controlled clinical trials are warranted to assess the extent of benefit and adverse effects of these medications in both the treatment and prevention of COVID-19.
